# Discovering Cooperative Relationships of Chromatin Modifications in Human T Cells Based on a Proposed Closeness Measure

**DOI:** 10.1371/journal.pone.0014219

**Published:** 2010-12-03

**Authors:** Jie Lv, Hong Qiao, Hongbo Liu, Xueting Wu, Jiang Zhu, Jianzhong Su, Fang Wang, Ying Cui, Yan Zhang

**Affiliations:** 1 College of Bioinformatics Science and Technology, Harbin Medical University, Harbin, China; 2 The Second Affiliated Hospital, Harbin Medical University, Harbin, China; University of Leuven, Belgium

## Abstract

**Background:**

Eukaryotic transcription is accompanied by combinatorial chromatin modifications that serve as functional epigenetic markers. Composition of chromatin modifications specifies histone codes that regulate the associated gene. Discovering novel chromatin regulatory relationships are of general interest.

**Methodology/Principal Findings:**

Based on the premise that the interaction of chromatin modifications is hypothesized to influence CpG methylation, we present a closeness measure to characterize the regulatory interactions of epigenomic features. The closeness measure is applied to genome-wide CpG methylation and histone modification datasets in human CD4+T cells to select a subset of potential features. To uncover epigenomic and genomic patterns, CpG loci are clustered into nine modules associated with distinct chromatin and genomic signatures based on terms of biological function. We then performed Bayesian network inference to uncover inherent regulatory relationships from the feature selected closeness measure profile and all nine module-specific profiles respectively. The global and module-specific network exhibits topological proximity and modularity. We found that the regulatory patterns of chromatin modifications differ significantly across modules and that distinct patterns are related to specific transcriptional levels and biological function. DNA methylation and genomic features are found to have little regulatory function. The regulatory relationships were partly validated by literature reviews. We also used partial correlation analysis in other cells to verify novel regulatory relationships.

**Conclusions/Significance:**

The interactions among chromatin modifications and genomic elements characterized by a closeness measure help elucidate cooperative patterns of chromatin modification in transcriptional regulation and help decipher complex histone codes.

## Introduction

Complexity and specificity of transcriptional control has long been the subject of intense research. Epigenetics is the study of biological outputs that are not defined by static genome sequences [1]. Histone modification and DNA methylation are the best known examples of epigenetic regulation. Recently data has helped shed light on the role of epigenetic modifications in transcriptional regulation [2]–[4]. Histone modifications play a significant role in epigenetics and can dynamically influence gene transcription [5]. Many types of histone modification are known to act on nucleosomes, but only a few of them have a defined function in genomic regulation. In addition, chromatin modifications often function in a cooperative way to increase regulatory complexity. Histone modifications have been shown previously to be one mechanism of modulating transcription factors (TFs) and transcriptional control [6], [7].

CpG methylation is the major covalent DNA modification in mammals and is another important epigenetic mechanism. DNA methylation is strongly linked to particular genomic elements. Several lines of evidence indicate that CpG islands (CGIs) generally repel CpG methylation, which is quite different from the bulk genome, especially genomic repeats where most CpGs are methylated [8]–[10]. Promoters may not contain CGIs, even though they may overlap significantly. Many possibilities have been proposed to account for the role of DNA methylation in transcription. One widely supported theme is that DNA methylation can impede TF binding to specific genomic fragments [11], [12].

Covalent modifications of histone tails, such as methylation and acetylation, contribute to the dynamic regulation of transcription [5], [13]–[15]. The *cis*-regulation of transcription by a large number of combinatorial histone modifications is called the histone code [16], [17]. A histone modification may colocalize with other modifications and may even be on the same histone tail. Although DNA methylation and histone markers are in different epigenetic layers, and both are regulated via enzymatic mechanisms [18]–[20], it is relatively straightforward to explore their interaction given that histone modification and DNA methylation often colocalize to influence each other. It has been suggested that TF cooperativity is dependent upon chromatin modifications [21], which prompted us to investigate cooperative signatures of epigenomic and genomic elements.

Several experimental studies have confirmed chromatin interactions [22]–[24]. Generally, these studies have suffered from being small scale and limited in the number of specific genomic loci examined. For example, a recent study identified a novel mechanism of DNA methylation in gene activation [25], quite different from the general repression mechanism. In addition, advances in experimental approaches have enabled high-throughput sequencing and genome wide studies to identify epigenetically regulated patterns [26]–[32]. The genome-wide characterization of epigenomic marks and genome-epigenome cooperativity by chromatin immunoprecipitation followed by massively parallel sequencing is therefore feasible. The resolution and genome-wide scale of these data enable the comprehensive investigation of regulatory patterns beyond CGIs and promoters, and more towards uncharacterized regions. In particular, the available data facilitates investigation of the chromatin modification landscape in functionally unknown regions and consequently can provide a more comprehensive view of biological interactions.

Bayesian network inference can identify regulatory networks. Edges in a Bayesian network can represent causal relationships. In this study, we used the WinMine package to infer chromatin regulatory relationships, as the algorithm in the package improves the original Bayesian network algorithm to distinguish compelled from reversible edges. Previous studies have demonstrated the usefulness of Bayesian networks for reconstructing regulatory networks [33], [34]. Yu et al. inferred the first epigenetic regulatory map of histone modifications and gene expression [34]. In their study, a Bayesian network proved to be an ideal tool for inferring regulatory relationships at 1.2, 2 and 4 kb size windows from ChIP data. Although we also use a Bayesian network, our approach is fundamentally different. We discovered regulatory chromatin modification relationships from derived feature modules using a Bayesian network based on a novel profile-based measure, called the closeness measure (*CM*). The *CM* is designed to capture influential effects of specific chromatin domains on CpG methylation. Computationally, the *CM* measure is based on the premise that cooperativity among epigenomic elements can affect the local methylation status at a specified, and nearby CpG loci if they overlap in terms of genomic position. To consider the genome-epigenome interaction, we selected CGIs, DNA repeats, and promoters, as representative of genomic elements together with other chromatin elements to construct a Bayesian network. DNA methylation was considered as the phenotype to infer the cooperativity among chromatin modifications. Intuitively, the closer a cytosine (within a putative chromatin domain) is around the center of the domain, the more influence the domain imposes on the cytosine, potentially cooperating with other epigenomic elements to influence DNA methylation. Therefore, the assumption is that chromatin interaction can be inferred from the methylation influences. We observed DNA methylation to have very limited regulatory roles. For clarity, we assume that CpGs are considered to be influenced by chromatin features and any features have not influences upon CpGs. Both “closeness” and “distance” can be used to characterize the interaction of chromatin modifications upon CpG loci, where the distance is proportional to the closeness. Given the contrasting genomic resolution of chromatin domains and single CpG loci, the *CM* is more suitable for quantifying their relationship than the distance measure.

**Figure 1:**
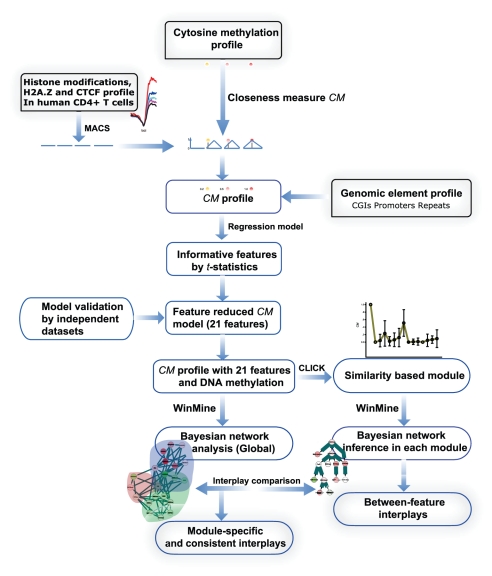
Study overview.

In this study, high-throughput DNA methylation and chromatin modification data processed by the proposed *CM* were assembled to profile 31,237 loci. To reduce the false positives, only features significantly associated with methylation as characterized by the *CM* regression model were kept. To find regulatory networks that associated with distinct chromatin patterns, we performed an unsupervised homogeneity based cluster analysis to obtain nine functional feature modules. Further investigation revealed that these modules were associated with distinct levels of gene expression and dominant biological functions. In the regulatory networks of the nine modules, DNA methylation and genomic elements are present only in specific modules, implying that they are not necessarily common regulatory initiators. Frequent interactions are considered consistent regulatory patterns. Our studies find many regulatory and cooperative chromatin modifications that have not been characterized experimentally. Finally, novel relationships were validated by partial correlation analysis. Data from this study and similar efforts help establish an epigenomic regulatory landscape and can be used as a reference by other studies and projects, such as The Chromatin Protein Discovery Project (CPDP). The workflow of this study is summarized in Figure 1.

## Materials and Methods

### Datasets

A small number of chromatin modifications have been confirmed to have cooperativity, and many relationships remain to be discovered. To obtain a better understanding of the regulatory and cooperative patterns of epigenomic and genomic elements in CD4+ T cells, we downloaded and analyzed various datasets. Firstly, we obtained publicly available ChIP-seq data for chromatin modifications, including histone modification, a histone variant H2A.Z and two transcription factor (CTCF and PolII) in human CD4+ T cells [27], [28], and transformed the number of tags for each data to putative chromatin domains by MACS from Zhang et al. [35]. The CpG methylation data in CD4+ T cells is available from the Human Epigenome Project (HEP) [36], where the direct Sanger sequencing of bisulfite-converted DNA was used to generate a CpG methylation landscape across human chromosome 6, 20, and 22. The methylation levels of CpG loci were averaged by ‘variation’ identifiers, and records of technical controls were discarded. The original genomic positions (hg17) of CpG loci were changed to genome assembly hg18 by the GALAXY server [37]. At last count, a data profile of 31,237 CpG loci was available (Table S1). The average methylation level is shown as the response in the regression model and used for linking epigenomic and genomic elements. The genomic distribution for the loci is presented in Table 1. We then linked the CpG loci in the DNA methylation profile data with chromatin modifications and genomic elements using the *CM* measure.

**Table 1:** Genomic distribution of CpG loci in HEP data for each chromosome.

Gene association class	Number (%)			
	Chromosome 6	Chromosome 20	Chromosome 22	Sum
# of CpG loci (%)	9757 (31.24)	5172 (16.57)	16308 (52.21)	31237 (100)
Promoter	4856 (49.77)	519 (10.03)	5647 (34.63)	11022 (35.29)
CGIs	5962 (61.10)	1055 (20.40)	6792 (41.65)	13809 (44.21)
Repeat	220 (2.25)	134 (2.59)	475 (2.91)	829 (2.65)
TSS[-10k, -1k]	1511 (15.49)	475 (9.18)	1908 (11.70)	3894 (12.47)
TSS[-1k, 0k]	2795 (28.65)	243 (4.70)	3360 (20.60)	6398 (20.48)
5′UTR	1094 (11.21)	106 (2.05)	1200 (7.36)	2400 (7.68)
Exon	3121 (31.99)	220 (4.25)	4996 (30.64)	8337 (26.69)
Intron	2797 (28.67)	1940 (37.51)	4566 (28.00)	9303 (29.78)
3′UTR	62 (0.64)	39 (0.75)	551 (3.38)	652 (2.09)
TES[0k, 1k]	291 (2.98)	10 (0.19)	317 (1.94)	618 (1.98)

In addition, three genomic markers (promoters, CGIs and repeats) were included in the *CM* data profile to understand the functionality of genomic elements and further uncover genome-epigenome interactions. The genomic features include the CGI annotation from CpGcluster [38], the repeat feature downloaded from UCSC (RepeatMasker feature), and the promoter feature defined by [−1k, 0.2k] around transcription start sites (TSSs).

### The closeness measurement for identifying cooperative chromatin interactions

**Figure 2:**
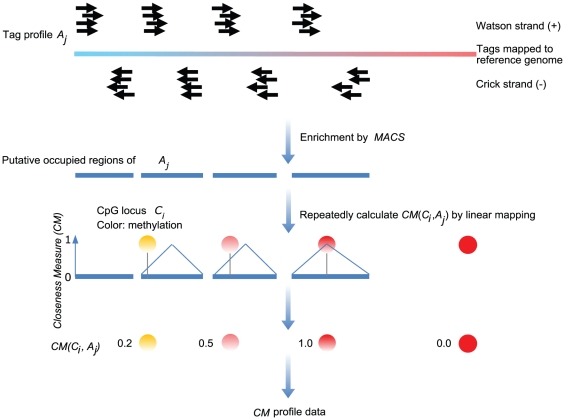
The workflow of the proposed distance measure.

Though epigenomic interactions have only recently been proposed, increasing evidence supports this hypothesis [39]–[41]. The *CM* is proposed to identify the interaction of epigenomic features based on their interactions with DNA methylation. The *CM* measure is based on the premise that chromatin modifications are combinatorially linked to DNA methylation [18]. The computational workflow of the *CM* is illustrated in Figure 2. The *CM* is applied to all types of putative chromatin modification domains. Conversely, the hidden interaction can be inferred from the *CM* data profile by treating the methylation status as the interaction indicator.

All 41 ChIP-seq chromatin features were processed by MACS [35] to detect putative chromatin domains of chromatin features. We first downloaded the ChIP-seq data sets [27], [28] and then processed them with MACS using default parameters and *mfold*  = 10. Thousands of putative domains were mapped to specific chromatin modifications. Overlapping domains were combined into a unique domain.

The closeness measure is devised as follows. Let all features be denoted as *A_1_* … *A_j_* … *A_f-3_*, *A_f-2_*, *A_f-1_*, *A_f_*, where *f* is the feature number (*f* = 44) and *A_f-2_*, *A_f-1_*, *A_f_* represent three genomic features: promoters, CGIs and repeats, respectively. Except genomic features, all other features were quantified as the closeness of domain center against CpG loci by the *CM* measure (see reasons below). For a specific cytosine, *i*, its correlation with *A_j_* was denoted as a vector *CM_i_  = * (*CM_i1_* … *CM_ij_* … *CM_i_*_(*f-3*)_) where *CM_ij_* represents the closeness of cytosine *i* and feature *A_j_*. Let the putative chromatin domains in each feature profile *j* be (*R_1_* … *R_k_* … *R_Nj_*), the number of putative domains in each feature profile *j* be (*N_1_* … *N_j_* … *N_f-3_*), *m_j_* be the length of feature *R_k_* (Equation 1) and *N* to be the cytosine number (*N* = 31,237). The closeness measure *CM* (*C_i_*, *R_k_*) is defined below as a piecewise function:
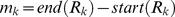
(1)

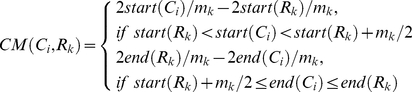
(2)where *start*(*R_k_*) and *end*(*R_k_*) are the start and end coordinate (0-based) of a putative chromatin domain mapped by ChIP-seq features and *start*(*C_i_*) and *end*(*C_i_*) are the start and end coordinate of a given CpG locus, *C_i_*. The boundary conditions in Equation 2 make the *CM*(*C_i_*,*R_k_*)  = 0, if none of the putative chromatin modification domains of *A_k_* overlap the specific CpG loci. The reasoning for this constraint is that CpGs beyond the outer edge of domains are not expected to be influenced by chromatin domains. The workflow of Equation 2 is illustrated in Figure 2. Equation 2 quantifies the relative position of a specific CpG locus around a putative chromatin domain centre, and normalizes by domain length (*m_k_*). The constant 2 appearing in every term of Equation 2 maps the range of closeness from [0, 0.5] to [0, 1]. This step makes the *CM* value comparable to the methylation level. The *R_k_* has to be previously merged if overlapped, so *CM*(*C_i_*,*A_j_*)  =  *CM*(*C_i_*,*R_k_*), if *CM*(*C_i_*,*R_k_*) ≠ 0. In Figure 2, we take four typical cytosines for instances.

### The discrete measurement for assessing the influence of genomic elements upon CpG methylation

It is unexpected that genomic elements (promoters, CGIs and repeats) exert closeness influences upon CpG loci. For genomic features, discrete values were coded, where *1* represents that the cytosine *i* overlaps the feature *A_j_* at least 1 bp, and *0* represents that the cytosine *i* does not overlap any domain in the profile *A_j_*, *j = f-2* … *f*.

To obtain a unified notation of the final profile, *CM*(*C_i_*,*A_j_*)  = 1 for overlap of *A_j_* (*j* = *f-2*, *f-1* and *f*) and *C_i_*; likewise *CM*(*C_i_*,*A_j_*)  = 0 for non-overlap of *A_j_* (*j* = *f-2*, *f-1* and *f*) and *C_i_*. As a result, the profile data is represented as the *N* by (*f*+1) matrix, in which each cell represents the *CM*(*C_i_*,*A_j_*) of cytosine *i* and feature *j* as well as a column of methylation status *M_i_* of cytosine *i*, where *j*<*f*+1 (Equation 3). The range of the profile is a 0–1 scale.
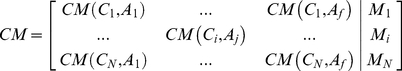
(3)


The *CM* profile data along with the information of the CpG loci and methylation status is presented in Table S1.

### Linear regression model and feature selection

Multiple linear regression functions in MATLAB (MathWorks, Natick, MA) were used to construct the regression model for the *CM* profile data derived by Equation (3). Coefficients *β_0_* and *β_j_* in Equation (4) was trained with the *CM* profile where the *M_i_* column was treated as the response variable *M*. The model parameter estimation was determined by 10-fold cross-validation. In each case, nine subsets were used for training and the remaining tenth subset for testing. The final Pearson correlation coefficient (Pcc) is the average of the ten cycles and is not limited to a specific subset of CpG loci.
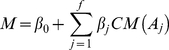
(4)


Informative features were determined by *t*-test (*p* = 0.001). The *t* statistics are defined as Equation (5).
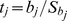
(5)where *b_j_* is the estimation of *β_j_* and 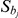
is the standard deviation of *b_j_*.

### Module detection and follow-up analysis

The CLICK clustering algorithm in the EXPANDER package was used to detect homogeneity based (epi)genomic modules [42]. Utilizing graph-theoretic and statistical techniques, CLICK is generally used to cluster gene expression profiles. Here, CLICK was used to classify CpG loci based on their *CM* profile.

To examine if the derived modules can be expected randomly, the values in *CM* profile were randomly swapped, keeping the same value distribution. We generated 100 such randomized datasets. For random datasets, the module number and homogeneity distribution is expected to be different from the real dataset.

We next examined the expression of genes associated with loci in these modules. The expression microarray (Affymetrix Human Genome U133 Plus 2.0 GeneChip array) from Schones et al. [43] provided the gene expression data from resting CD4+ T cells. The logarithmic profile derived from raw data was used, followed by quantile normalization (normalize quantiles function in R Affy package). The expression values for redundant Entrez Gene IDs were mapped by median probe values.

Specific regulatory functions are expected to group in specific modules. To demonstrate this, the enrichment of Gene Ontology (GO) Biological Processes (BP) terms was used to annotate the genes in modules by DAVID [44]. A modified Fisher Exact test proposed by DAVID identified significantly enriched GO terms within specific modules. The resulting *p*-value was corrected for false discovery rate (FDR) multiple hypothesis testing on the GO terms tested in each module. Only terms enriched by at least 1.5 fold over the average and EASE *p*<0.001 are shown.

To further assess intra-module gene function similarity, the mgeneSim function based on Wang's method in the GOSemSim (R package) was used to estimate semantic similarity of GO terms [45], [46]. Wang's method determines the semantic similarity of GO terms based on both the location of terms in the GO graph and their relation to ancestor terms. MgeneSim computes pairwise similarity scores for a list of genes. MgeneSim uses gene IDs as inputs, so we mapped the CpG loci in each module to get gene IDs (refgene_getnearestgene in the CisGenome package). MgeneSim automatically removes genes without annotations. Finally, we calculated the median gene similarity results together with the standard deviation based on the output of mgeneSim.

To show the genomic distribution of CpG loci in the methylation dataset from the HEP project, gene structures of these loci (i.e. exon) were annotated by function using refgene_getlocationsummary in the CisGenome package [47].

### Bayesian network inference

We used the GES tool in the WinMine Toolkit (http://research.microsoft.com/en-us/um/people/dmax/WinMine/tooldoc.htm) to build Bayesian networks based on the *CM* profile. Each chromatin feature together with its methylation status in the *CM* profile was split into two classes based on equal frequency. In principle, the Bayesian network is well illustrated in a recent paper [34].

All together, two types of Bayesian networks were mapped. One is a global network, which is without module partition. The other one is a module-specific network. The global network reflects the overall regulatory relationships and can be compared with module-specific networks.

Network topology was analyzed by two Cytoscape plugins (ClusterViz and RandomNetworks). First, the EAGLE algorithm in ClusterViz was used to identify network modules, with CliqueSizeThreshold  = 3 and OutputThreshold  = 2. To check whether the real network was a random event, the RandomNetworks plugin randomized existing networks and compared the network features of existing networks to randomized networks. Key parameters in RandomNetworks include Num shuffles (Number of shuffling), set at 5000, and Rounds to Run (Number of Randomizations to Perform), set at 5000.

Using 100 random datasets generated by module randomization, network construction was repeated 100 times for the global network and module-specific networks to determine whether regulatory relationships are generated by random data.

## Results

### 21 chromatin modifications and genomic elements are selected as informative features

The *CM* was used for linking chromatin modifications and DNA methylation to generate the *CM* profile. We then used a linear regression model to select potential features as candidates that may function combinatorially in human CD4+ T cells. The selected features are expected to have individual closeness influence on cytosine methylation. From the estimated regression coefficients and *p*-values in the trained model, including all 44 (epi)genomic features and methylation levels (response variable), we obtained 21 features (*p*<0.001) that appeared to influence methylation and selected those features for further analysis (Table 2).

**Table 2:** Significant features in the feature-reduced model, together with corresponding *P*-value.

Feature	*P*-value*	Feature	*P*-value*
CGI	0.00	H3R2me1	0.00
Promoter	0.00	H4K20me1	0.00
CTCF	4.44E-16	PolII	1.45E-6
H2A.Z	4.25E-11	H4K91ac	4.97E-9
H2BK5me1	0.00	H2BK12ac	3.17E-11
H3K4me1	2.90E-5	H2BK20ac	6.04E-4
H3K4me2	0.00	H3K4ac	7.29E-9
H3K4me3	0.00	H3K9ac	8.94E-5
H3K36me3	0.00	H3K18ac	3.39E-6
H3K79me1	2.06E-4	H4K8ac	2.74E-12
H3K79me2	1.98E-8		

* *P*-value indicates the significance by *t* test.

To determine whether these features are sufficient to model CpG methylation, we created a feature reduced model (FRM) without insignificant features. We evaluated whether features are related to CpG methylation by determining Pearson correlation coefficient (Pcc) between modeled and measured methylation. As a result, there is a significant correlation (Pcc  = 0.891; empirical *p*<10^−6^) in the FRM. Random perturbation datasets (1,000,000) were generated from the *CM* profile, none of which yielded better Pcc than the constructed models. Significant features generated from the FRM and their coefficients and *p*-values are presented in Table 2 and Table S2, respectively. In contrast, there is a significant correlation (Pcc  = 0.877; permutation empirical *p*<10^−6^) for models generated using all features. These results suggest that only some chromatin modifications are significant enough to impact the CpG methylation model. Unexpectedly, when the repeat feature should be excluded from the FRM (*p* = 0.35).

We next evaluated how the FRM performs with independent datasets by firstly using another publicly available CD4+ T cell methylation dataset generated using the“Illumina GoldenGate Array for Methylation”, designed for sequencing up to 1,536 loci [48]. After filtering, 571 loci that do not overlap with the training data were left for validation. Though not excellent, the predicted methylation status by the FRM correlated with the measured status, although not as strongly (Pcc  = 0.602; empirical *p*<10^−6^).

A MeDIP-chip experiment is quite different from a bisulfite-converted DNA experiment in principle, and is expensive when used to generate genome-wide maps at a high resolution. However, there is a MeDIP-chip genome-wide dataset containing 345,274 regions (approximately 100 bp) generated from CD4+ T cells by Rakyan et al. [49]. We used this dataset to for our evaluation. Since the resolution of their data is significantly lower than the training dataset used in the FRM, we extracted centrally located CpG as a stand-in for each region. Nearly all such CpGs do not overlap with CpGs in the FRM training dataset, decreasing the possibility of overstating the prediction accuracy. Unexpectedly, we observed a high correlation between the estimated and observed methylation level (Pcc  = 0.941). Interestingly, when we swapped the role of training and testing datasets, we observed a similar yet lower correlation (Pcc  = 0.876). Therefore, the classifier based on the MeDIP-chip data is less accurate than the HEP data, most likely due to the lower resolution of MeDIP-chip data, even though the number of regions is 10-fold more than in the HEP dataset. We believe that predictions based on the FRM can achieve reliable predictions even when there is a low number of training CpG loci, based on this analysis.

We have demonstrated the effectiveness of a *CM* model incorporating information from 21 features that influence DNA methylation based on *CM* measure by HEP and independent datasets. We proceeded with the variables of interest as determined using the FRM to further explore cooperative and regulatory relationships.

### Modularity of epigenomic and genomic elements based on functional analysis

**Figure 3:**
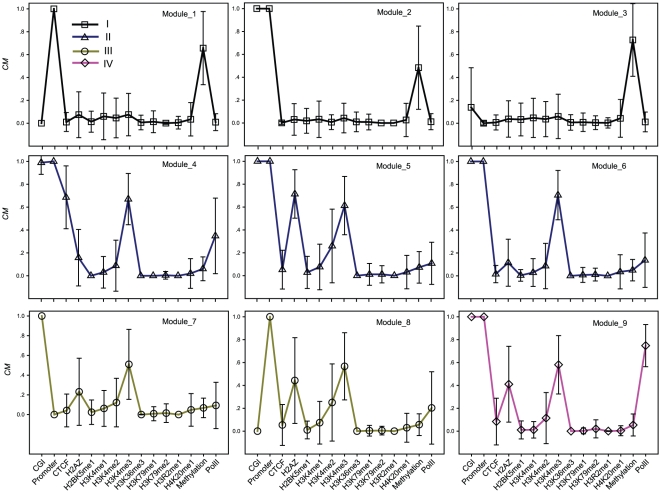
The homogeneity based clustering. Nine modules with distinct (epi)genomic patterns, involving significant features based on regression analysis. X-axis: the significant epigenomic features from Table 1, excluding histone acetylation marks. The order of features is arbitrary. Y-axis: the closeness of features around CpG loci as measured by *CM*. Symbol height represents the median *CM* value. Error bars represents standard error. The number under the Module_*i* (*I* = 1, …,9) is the count of CpG loci in Module_*i*, The specific proportion of each module is shown in Figure 4A. Modules are roughly classified into four meta-groups according to genomic elements and PolII patterns, as indicated by the four colors.

We hypothesized that similar (epi)genomic patterns are associated with common regulatory functions. To identify (epi)genomic patterns in an unbiased, genome-wide approach, we examined all loci using a feature selected *CM* profile to find over-represented chromatin modification patterns. Modules were grouped by the cluster algorithm CLICK, which does not make prior assumptions and avoids potential biases of the relatively limited CpG loci available here since they do not require pre-determined seeds [42]. Using this approach, nine CpG loci pattern modules were obtained (Figure 3).

It is useful to explore how about the hidden patterns of (epi)genomic elements may cooperate to influence gene regulation. Loci in different modules have quite distinct genomic distributions (Table S3). For example, 74%, 68% and 65% of loci in Module_4, Module 5 and Module_8, respectively, are located 1k upstream of gene TSSs. In contrast, very few loci are located in the region for Module_3 and Module_7. To exclude the influences of genomic elements and to explore the effects of chromatin signatures in transcription, we classified these modules into four meta-groups (Figure 3). All three modules in Group I show elevated levels of methylation and correlate with low PolII expression. Group II contains an over-representation of CGI, promoters, H2A.Z and H3K4me1/2/3 modifications, low levels of DNA methylation, and intermediate PolII levels. Group III includes elevated H2A.Z and H3K4me1/2/3 and low CTCF levels. In addition, the proportion of loci in CGI and promoters are reversed in Module_7 and Module_8. Group IV contains only Module_9 and shows the highest PolII level and resembles Module_8, except that Module_8 is CGI independent. Based on the above observations, we sought to further explore whether these modules are functional and whether they differ in gene expression.

**Figure 4:**
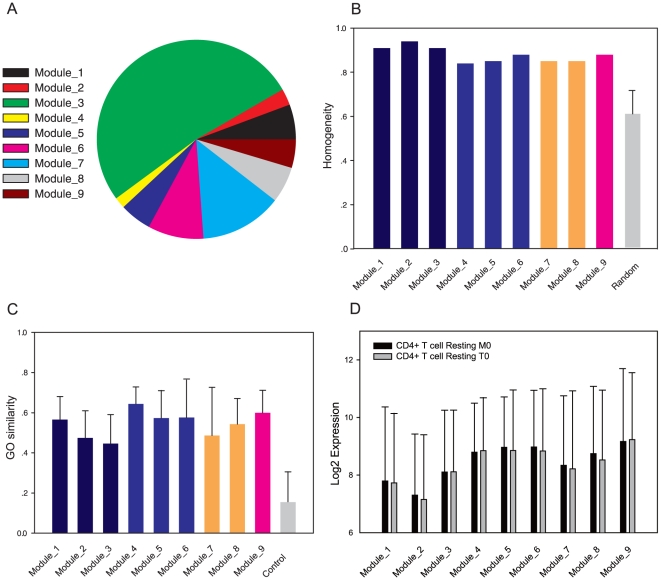
The loci distribution and function evaluation for all modules. (A)The proportion of CpG loci in Module_*i*. (B) The homogeneity in each module as reported by the CLICK algorithm in the EXPANDER package. See the EXPANDER manual for details. The colors of bars are consistent with Figure 3. (C) The GO similarity of each module. The colors are consistent with Figure 3. Only Biological Process terms were used to calculate gene similarity. The similarity values standard deviation of each module is indicated as error bars, with one standard deviation in each direction. (D) The gene expression levels of 9 modules. The gene expression data is probed from resting T cells (M0 and T0 types). The bar height represents the median Log2 values of modules. The standard deviation of the Log2 values of each module is indicated as error bars with one standard deviation in each direction.

Figure 4A shows the loci number distribution across nine modules. The homogeneity representing the within-module pattern similarity is high in all modules, although the loci number in different modules varies (Figure 4B). We found that changing the order of rows in a *CM* profile does not alter the module number. Random profiles can only form modules with low homogeneity (Figure 4B) and when the module number is significantly larger than real data (23 in average, rank sum test *p*<10^−5^). Histone acetylation markers were not shown, as it seems that they have not module-specific signatures (data not shown). Only a small fraction of CpG loci (195, 0.6%) is excluded by CLICK without clustering, suggesting a limited module number for the chromatin signatures in a *CM* profile and that the (epi)genomic patterns are enumerable.

Genes possessing module-specific chromatin patterns would be expected to have expression differences, as suggested in previous studies [28], [50]. The median probe expression of each module is shown in Figure 4D. It is intriguing that the gene expression levels are significantly distinct between meta-groups (Dunn's Method, *p*<0.001), but are often insignificant within meta-groups (data not shown), implying the partition of modules is reasonable. For random permutation datasets, no significant differences were found between modules in terms of gene expression.

We next evaluated our approach for how effectively to discriminate gene function. We used the GOSemSim package from CRAN (http://cran.r-project.org) to evaluate intra-module gene function similarity. Only “Biological Process” terms were evaluated. We used the 195 loci abandoned by CLICK as the control group. As shown in Figure 4C, the gene function similarities for nine modules are significantly higher than control (rank sum test *p*<0.01), consistent with there being similar biological function within modules. We performed a functional enrichment to discover the dominant gene function in different modules. The enrichment analysis indicates that genes involved in chromatin assembly are most enriched in Module_1 (5 terms), Module_2 (4 terms) and Module_8 (7 terms). In addition, protein modification processes are enriched in Module_5 (2 terms) and Module_9 (3 terms). Genes involved in biogenesis and metabolic processes are enriched in Module_3 (5 terms), Module_7 (1 term), Module_8 (3 terms) and Module_9 (2 terms). Development related terms are only enriched in Module_3 (1 term) and Module_7 (6 terms). The significant terms for each module are presented in Table S4. These results indicate that the modules identified are grouped by specific biological function and are consistent in terms of gene expression and function.

### Bayesian network inference from chromatin feature modules

Although individual features may influence cytosine methylation, it is not clear whether and how they interact. To investigate the regulatory relationships among features, we evaluated the Bayesian network inference from the feature selected *CM* profile. The Bayesian network approach can demonstrate the dependency among features by maximizing joint conditional probability distributions. We used the WinMine package because it improves the original algorithm and keeps only compelled but not reversible edges (compelled edges correspond to causal relationships, while reversible edges might be merely correlated).

**Figure 5:**
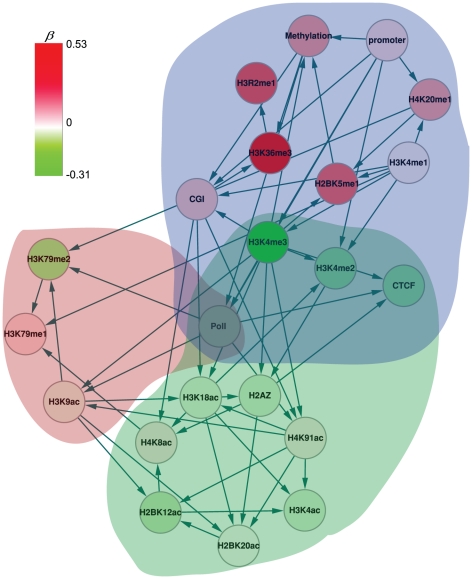
The global Bayesian network inferred from the complete *CM* profile. The red, blue and green circles represent different groups. The node color is mapped by the color key in the left top corner, representing the coefficients of the regression model (Table S2).

**Figure 6:**
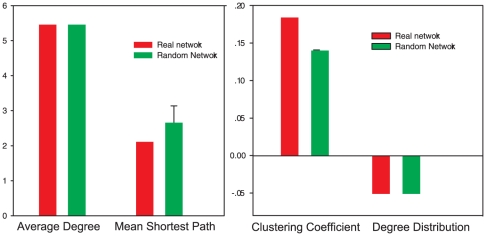
Network metrics for the Bayesian network. Network manipulations were performed in Cytoscape, and the clustering coefficient, average degree, power exponent in degree distribution, and mean shortest path were derived by the Cytoscape Random Networks plug-in.

A global inferred network was generated as a control for module-specific relationships, prior to generating the module-specific inference. The regulatory network is defined by three network groups (distinguished by three colors), including significant features and DNA methylation (Figure 5). The figure suggests that chromatin modifications and genomic features form a highly connected regulatory network, and that certain features co-function in concert with other specific features for activation or repression. It also appears that genomic features are correlated with chromatin modifications, such as the relationship of H3K4me3 with CGI [51]. Previous studies found that H3K4me3 is catalyzed by H3K4 methyltransferases that are recruited by PolII [52]. Interestingly, PolII tends to bind at CGIs [53]. Compared to random networks, these networks demonstrate significant resistant to perturbations (Figure 6). It is noteworthy that the average degree distribution is essentially the same between real and random networks, for the random network maintains the same number of edges as the real network. Topologically, the networks tend to be tolerant of small perturbations. In contrast, none of the regulatory relationships were in all random datasets.

**Figure 7:**
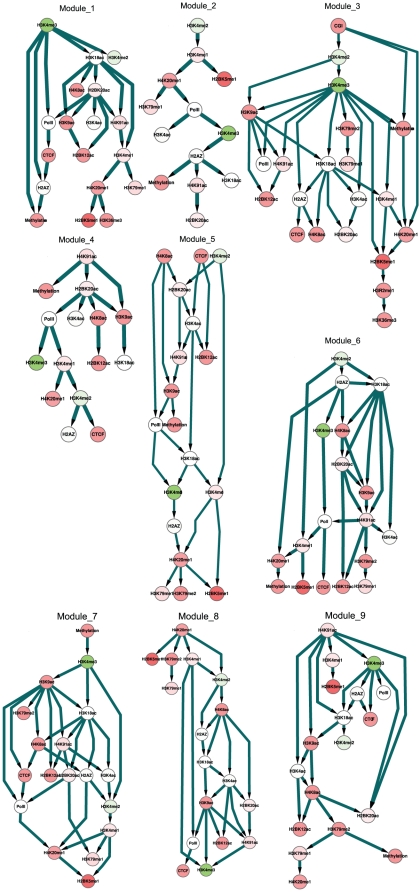
The Bayesian network inferred from nine modules. The node color is mapped by the color key in the left top corner, representing the coefficients of the regression model (Table S2). All node color is mapped by the color key (Figure 5).

Because distinct modules reveal distinct transcriptional, gene function and genomic signatures, we were interested in investigating module-specific regulatory relationships. To this end, we performed the Bayesian network inference for each module as shown in Figure 7. We used 100 random datasets of each module to perform network inference but none of the regulatory relationships were present in all modules. Similar to the global network, module-specific networks generally exhibit significantly higher clustering and shorter mean shortest path than would be expected, except for the simple networks of Module_2 and Module_4 (data not shown).

Comparing the global and module-specific networks enables evaluation of the relationship between DNA methylation, genomic elements, and chromatin modifications. It is worth noting that promoters are absent in every module and CGI are present only in Module_3, where they initiate subsequent regulation relationships (Figure 7). In contrast, regulatory relationships involving genomic elements are present more in the global network (Figure 5). These results imply that CGI provides only the baseline for chromatin interactions. For all modules, DNA methylation is regulated by H2A.Z, H3K4me3, H4K20me1, H3K79me2, H4K91ac, H3K9ac and CGI. Only H2A.Z [54], H3K4me3 [55] and CGI have been documented to be related to DNA methylation. In addition, H3K4me3 indirectly regulates DNA methylation via H2A.Z (Module_1 and Module_2). We then explored the regulatory role of DNA methylation upon other features and found that DNA methylation has a regulatory role only in Module_7. In contrast, DNA methylation is the regulatory terminator in Modules_1,2,4,5,6,9. In Module_8, DNA methylation is absent. Based on these results, it is reasonable that this work only considers DNA methylation as a stable phenotype, which underlies the closeness measure. These studies further suggest that (epi)genomic elements are correlated in the global and module-specific networks. These regulatory relationships may provide insights into the biological function of epigenomic and genomic elements, wherein the inferred relationships could be served as reference for further studies.

### Discovery and validation of cooperative chromatin modifications *in silico*

**Figure 8:**
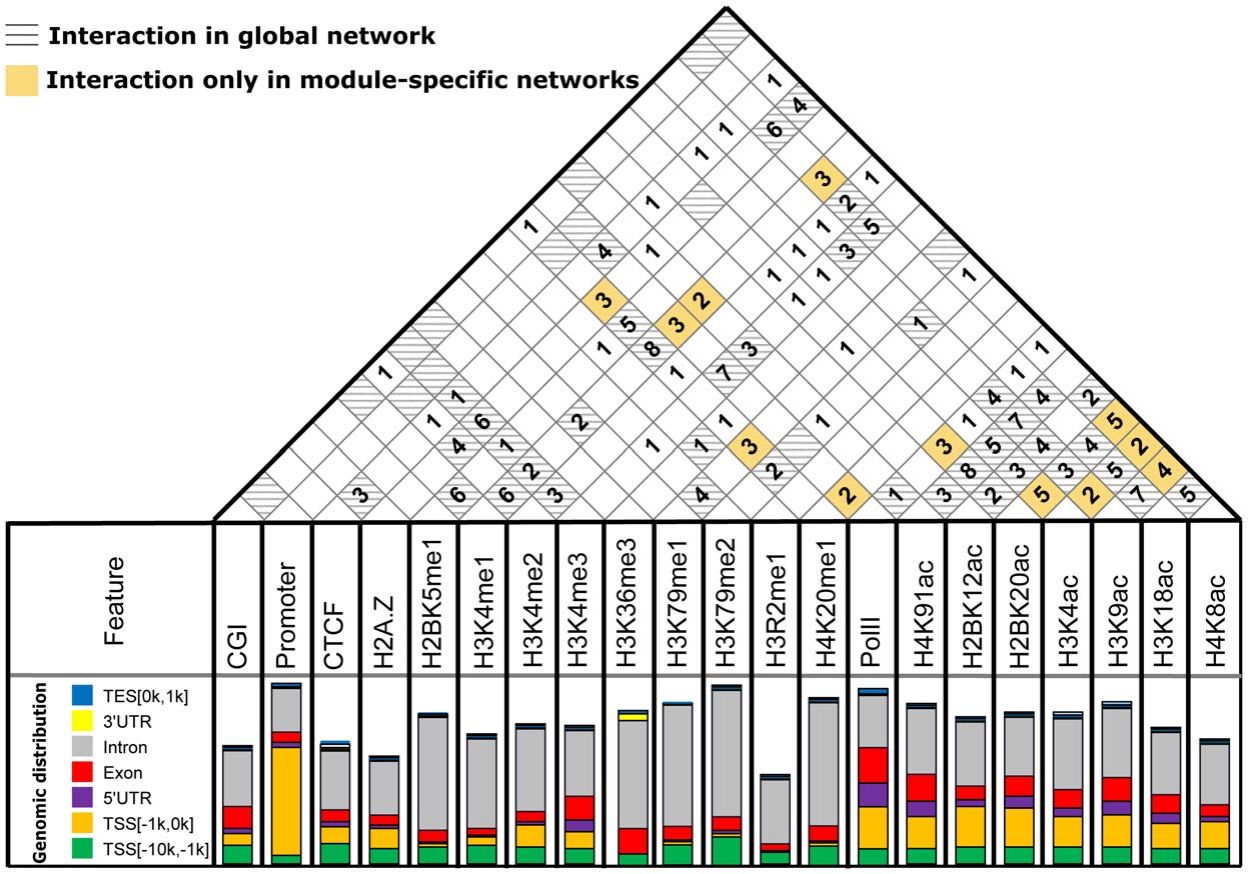
The between-feature interactions from module-specific Bayesian inference. The top panel shows the occurrence of between-feature interactions in nine Bayesian network maps, where each cell represents one specific interaction type and the number within each cell represents the number of occurrences in all nine modules. The yellow color marks the module-specific and significant interactions discovered at least in two modules. The hatch marks the regulatory relationships only in the global network (Figure 5). The number in the square counts the occurrence of between-feature interactions in nine modules. The bottom panel shows the genomic distribution of each feature.

It is informative to show only undirected feature interactions in regulatory networks when exploring genomic and chromatin cooperativity. Cooperative relationships are also easier to detect than regulatory relationships experimentally, for it is difficult to distinguish causal from correlated relationships. In Figure 8, the top panel shows the occurrence of pairwise interactions in a Bayesian network where the recurring between-feature interactions are considered robust against perturbation. Such interactions (occurrence >1) are presented and sorted by descending order in Table S5. Frequently occurring relationships are considered as vital regulatory relationships. Module-specific regulatory relationships are shown in yellow (Figure 8). The global network serves as a control (Figure 8). We noted some interactions, such as H3K4me1-H4K20me1, H3K4me3-PolII, are prevalent in nine modules. Moreover, 40 interactions (74%) in the global network are also present in Figure 7, suggesting the robustness of (epi)genomic interactions. However, the interactions discovered from each module are a little different from the global network, suggesting many interactions are module-specific and we can obtain module-specific interactions. In particular, most relationships involving genomic elements are present only in the global network, which is considered to have cross-module regulatory roles that may not be discovered by module-based Bayesian networks.

Only a few prioritized between-feature interactions have been directly or indirectly reported [23], [24], [52], [56]–[61] (Table S5). The available evidence indicates that several cooperative chromatin modifications characterized experimentally demonstrate cooperativity in our analysis, validating the use of the mining process to identify potential cooperativity. For example, in yeast, absence of H2A.Z is correlated with reduced H3K4me3 level [24]. In addition, Set1, the H3K4 methylase, is recruited by PolII at the 5′ ends of active mRNA coding regions in yeast [52]. The H3K79 methyltransferase Dot1L-deficient ES cells show reduced levels of H4K20me at centromeres and telomeres [23], [52]. However, many of the relationships in the regulatory networks have not yet been reported, generating experimentally testable hypotheses. Though no experimental evidence has been reported for the interaction of H4K20me1 and PolII, H4K20me1 and H2BK5me1, H3K4me2 and H2A.Z, H2BK5me1 and H3K4me1, or H2A.Z and H3K4me3, these relationships were consistent with the inferred regulatory network from Yu et al. [34]. This observation suggests that our results are biologically reasonable. The inferred cooperative interactions differ a little from Yu et al., possibly because the histone acetylation marks and genomic elements are cooperative and alter the regulatory network. Relationships discovered in CD4+ T cells are supported by literature reports in other species and tissues. Perhaps the regulatory pathways in different cells share a degree of conservation, just as the histone code seems consistent in diverse cells. Therefore, the pipeline we used may also provide clues for chromatin regulatory mechanisms in other cells.

Very few chromatin interactions have been validated so far (particularly not genome-wide) and there is no “gold standard” to estimate the performance of our approach. In addition to support contained in the published literature, the cooperative relationships relating to chromatin modifications are indirectly supported by partial correlation analysis based on experimental data. We therefore performed the partial correlation analysis of chromatin modifications and used the “causal” partial correlation coefficient to provide direct reference for novel regulatory and cooperative relationships that may be true *in vivo* (see details, Table S6). To avoid potential biases, ChIP-seq chromatin modifications from two cell types were used (GM12878 and Hsmm). To examine if the correlation was random, 100 random permutation tag profiles were generated to provide a control. The results showed that all discovered relationships involving the available histone modifications (Table S2) have significantly higher partial correlation coefficients than others (Wilcoxon rank sum test, *p* = 0.04 for GM12878 and *p* = 0.05 for Hsmm) (Table S6) and higher than random (*p*<10^−4^).

From the methodology perspective, our analysis pipeline integrates features (most ChIP-seq) and CpG methylation data to obtain the interactions between features and adds a powerful and much-needed tool for examining regulatory relationships both for well-studied features and for less-studied features. While this analysis was performed only for human CD4+ T cells, it can be readily extended to all cell types and conditions. The Chromatin Protein Discovery Project that was started in 2008 aims to generate a regulatory map for a set of candidate chromatin proteins in Drosophila. The project should help understand chromatin regulation by identifying dozens of novel components and their interactions. The candidate proteins used in the project are selected by computational prediction. We believe that our approach and similar efforts that target candidate chromatin components and interactions would be useful for further elucidation of chromatin regulation. We anticipate such efforts will be helpful for analyzing how transcriptional regulation is encoded and re-programmed.

## Discussion

To understand mechanisms of epigenetic regulation, it is imperative to investigate the cooperative nature of chromatin modifications and genomic elements. Here, we reported a regulatory inference model of epigenomic and genomic interactions. This model can predict many novel chromatin interactions, and the module-based regulatory networks provide insights into the relationships of (epi)genomic patterns, chromatin interaction and genomic function. For example, genomic loci in Module_7 are associated with development. Interestingly, DNA methylation has regulatory roles only in Module_7. Therefore, the results may also help identify CpG loci associated with particular functions.

Previously, few computational approaches for studying genome-wide epigenetic regulation have focused on the discovery of functional chromatin regulatory relationships and cooperativity. ChromaSig developed by Hon et al. is one algorithm that can find recurring chromatin signatures based on histone modification profiles [62]. Similar to our approach, ChromaSig can also find chromatin patterns without relying explicitly on the expected cluster number. Previous approaches such as ChromaSig overlook the regulatory roles of CpG methylation and can only evaluate epigenetic patterns without considering genomic elements. Therefore, a tool that can analyze CpG methylation, genomic and epigenomic data is still needed. The lack of such tools is partly caused by the poor biological interpretation and partly by inefficient bioinformatics algorithms. Previous studies carried out window-based approach (tag count within a fixed-size window) to model relationships among epigenomic features. Though this approach is also applicable to deriving biological relevance [50], the window-based measures tend to fail in two types of cases. The first is where a tag number within a specific window is very large, e.g. over 1000, which is often observed in large-scale datasets and in a human histone modification database developed by Zhang et al. [63]. However, the regression model with our proposed measure does not bias towards such regions. Though cutoffs for trimming large values can make up for the extreme values, it is not biologically plausible to do so and may affect model parameter estimation and regulatory pattern inference. The second is where the window-based method is most suitable for intensity data quantified in a large region, but is not suitable for analyzing CpG methylation, although Yu et al. showed that window size changing from 1.2 to 4 kb does not affect Bayesian network construction [34].

The success of our proposed approach also depends on the correct identification of chromatin domains from ChIP-seq data. Genomic regions mapped by specific histone modifications may fail to be sequenced by ChIP-seq or to form peaks, in which case they would be overlooked by peak calling tools. To estimate the reliability of chromatin domain calling tools, we compared a list of true histone modification domains by different ChIP-seq peak finding algorithms. As a result, there is a significant overlap among different algorithms, though it is noteworthy that some other algorithms bias towards longer or shorter regions [64]. In fact, MACS is a balancing algorithm with detection sensitivity and specificity (data not shown). However, if specific genomic loci are free of any histone modifications, the analysis based on the chromatin modification profile would not work for the *CM* measure and even the window-based approaches. Specially, neither H3K9 nor H3K27 methylation marks are included in the FRM. It is possible that few CpG loci selected for methylation sequencing are proximal to any H3K9 or K27 enriched marks and even more possible that these repressive methylation marks cooperate with other unanticipated markers to influence DNA methylation. Therefore, it is possible that some expected yet missing interactions may represent an underestimate by the not so much data.

Previous studies have identified the common histone-modifying enzymes for acetylation marks on histone tails [65], [66]. There is substantial data indicating that histone acetylation marks directly influence DNA methylation [67]. All coefficients of acetylation marks are close to 0.05, though their interactions are significant (Table S2). In Figure 7, the direct regulatory relationships of DNA methylation and acetylation marks occur only in Module_4 and Module_5. Therefore, their interactions are implied as a local process, and histone acetyltransferases may not interact with DNA methylation directly but be correlated via other factors.

Though there are 31,237 distinct CpG loci with high-throughput DNA methylation and chromatin modification data in the *CM* profile used for model construction and regulatory relationship inference, it is not quite enough for generating robust results. The testing MeDIP-chip dataset containing more CpGs show that the HEP methylation data is well correlated with histone modifications. The chromatin regulatory relationships were supported by literature and experimental data. The relatively small amount of data does not bias the results. However, it is helpful to use larger scale data to test if the conclusions still hold when such data is available.

We defined a set of potentially influential methylation features by regression analysis. For simplicity, only significant features in the FRM are considered to contribute to the regulatory network and cooperativity of epigenomic marks. Further studies should extend this work to consider the insignificant features directly associated with significant features, but not influential in DNA methylation. It may also be useful to consider TFs as an important factor to account for transcriptional regulation, and ultimately have a more comprehensive regulation map.

## Supporting Information

Table S1 Summary of CpG loci used in this paper and the full profile data. This file contains genomic coordinates (hg18), methylation status, module association, and other genomic annotation in the *CM* profile data.
(3.74 MB TXT)

Table S2 Significant features used in this study, together with their corresponding β*_j_* defined in Equation 4 and *P*-value in the feature reduced model.
(0.04 MB DOC)

Table S3 Genomic distribution of CpG loci associated with nine modules.
(0.04 MB DOC)

Table S4 GO terms enriched in nine modules.
(0.12 MB DOC)

Table S5 Potential between-feature interactions (Occurrence >1).
(0.05 MB DOC)

Table S6 Partial correlation coefficients for chromatin modifications in GM12878 and Hsmm.
(0.05 MB DOC)
