# Supplementary material for: Discovering Cooperative Relationships of Chromatin Modifications in Human T Cells Based on a Proposed Closeness Measure
Source: PLoS One. 2010 Dec 3;5(12):e14219. doi: 10.1371/journal.pone.0014219 (PMC2997069; doi:10.1371/journal.pone.0014219)
Supplement: Table S2 — Significant features used in this study, together with their corresponding βj defined in Equation 4 and P-value in the feature reduced model. (0.04 MB DOC) [file pone.0014219.s002.doc]

Table S2 Significant features used in this study, together with their corresponding β*_j_* defined in Equation 4 and *P*-value in the feature reduced model.

| Feature | β*_j_* | *P*-value* | Feature | β*_j_* | *P*-value* |
| --- | --- | --- | --- | --- | --- |
| CGI | -0.12 | 0.00 | H3R2me1 | 0.38 | 0.00 |
| Promoter | -0.08 | 0.00 | H4K20me1 | 0.20 | 0.00 |
| CTCF | -0.07 | 4.44E-16 | PolII | -0.03 | 1.45E-6 |
| H2A.Z | -0.04 | 4.25E-11 | H4K91ac | 0.05 | 4.97E-9 |
| H2BK5me1 | 0.34 | 0.00 | H2BK12ac | -0.07 | 3.17E-11 |
| H3K4me1 | 0.04 | 2.90E-5 | H2BK20ac | 0.02 | 6.04E-4 |
| H3K4me2 | -0.08 | 0.00 | H3K4ac | -0.05 | 7.29E-9 |
| H3K4me3 | -0.31 | 0.00 | H3K9ac | -0.03 | 8.94E-5 |
| H3K36me3 | 0.53 | 0.00 | H3K18ac | -0.03 | 3.39E-6 |
| H3K79me1 | 0.07 | 2.06E-4 | H4K8ac | 0.06 | 2.74E-12 |
| H3K79me2 | -0.12 | 1.98E-8 |  |  |  |

* *P*-value indicates the significance by *t* test
