# Supplementary material for: Discovering Cooperative Relationships of Chromatin Modifications in Human T Cells Based on a Proposed Closeness Measure
Source: PLoS One. 2010 Dec 3;5(12):e14219. doi: 10.1371/journal.pone.0014219 (PMC2997069; doi:10.1371/journal.pone.0014219)
Supplement: Table S3 — Genomic distribution of CpG loci associated with nine modules. (0.04 MB DOC) [file pone.0014219.s003.doc]

Table S3 Genomic distribution of CpG loci associated with nine modules

| CpG loci association class | # of CpG loci (%) in Module_* | | | | | | | | |
| --- | --- | --- | --- | --- | --- | --- | --- | --- | --- |
| Meta-group | I | | | II | | | III | | IV |
| Module_ | 1 | 2 | 3 | 4 | 5 | 6 | 7 | 8 | 9 |
| # | 1761 | 826 | 16075 | 563 | 1601 | 2814 | 4144 | 1820 | 1438 |
| TSS[-10k, -1k] | 138(8) | 131(16) | 2456(15) | 13(2) | 85(5) | 131(5) | 722(17) | 135(7) | 59(4) |
| TSS[-1k, 0k] | 802(46) | 342(41) | 9(0) | 416(74) | 1082(68) | 1507(54) | 70(2) | 1184(65) | 895(62) |
| 5’UTR | 154(9) | 125(15) | 181(1) | 105(19) | 272(17) | 584(21) | 271(7) | 234(13) | 435(30) |
| Exon | 461(26) | 253(31) | 4194(26) | 177(31) | 390(24) | 842(30) | 932(22) | 469(26) | 551(38) |
| Intron | 362(21) | 107(13) | 5092(32) | 56(10) | 432(27) | 669(24) | 2026(49) | 396(22) | 144(10) |
| 3’UTR | 17(1) | 0(0) | 549(3) | 0(0) | 0(0) | 1(0) | 76(2) | 9(0) | 0(0) |
| TES[0k, 1k] | 345(20) | 79(10) | 2474(15) | 130(23) | 330(21) | 632(22) | 826(20) | 497(27) | 302(21) |
| TES[1k, 10k] | 401(23) | 112(14) | 2725(17) | 133(24) | 352(22) | 693(25) | 911(22) | 531(29) | 369(26) |
