# Supplementary material for: Discovering Cooperative Relationships of Chromatin Modifications in Human T Cells Based on a Proposed Closeness Measure
Source: PLoS One. 2010 Dec 3;5(12):e14219. doi: 10.1371/journal.pone.0014219 (PMC2997069; doi:10.1371/journal.pone.0014219)
Supplement: Table S4 — GO terms enriched in nine modules. (0.12 MB DOC) [file pone.0014219.s004.doc]

**Table S4 GO terms enriched in nine modules.**

|  | Term (Biological Process) | *P*-value | Fold |
| --- | --- | --- | --- |
| M1 | nucleosome assembly | 8.10E-06 | 7.64 |
|  | chromatin assembly | 1.01E-05 | 7.38 |
|  | protein-DNA complex assembly | 1.35E-05 | 7.05 |
|  | nucleosome organization | 1.54E-05 | 6.90 |
|  | chromatin assembly or disassembly | 1.82E-05 | 5.68 |
|  | DNA packaging | 6.30E-05 | 5.48 |
|  | immune system process | 1.33E-04 | 2.01 |
|  | cell communication | 4.59E-04 | 2.02 |
|  | cellular macromolecular complex assembly | 6.35E-04 | 2.77 |
|  | cell-cell signaling | 7.74E-04 | 2.14 |
|  | macromolecular complex assembly | 8.56E-04 | 2.05 |
| M2 |  |  |  |
|  | nucleosome assembly | 3.95E-04 | 12.59 |
|  | chromatin assembly | 7.36E-04 | 2.85 |
|  | protein-DNA complex assembly | 7.66E-04 | 2.82 |
|  | chromatin assembly or disassembly | 9.84E-04 | 2.77 |
| M3 |  |  |  |
|  | antigen processing and presentation | 3.34E-06 | 4.85 |
|  | antigen processing and presentation of peptide antigen | 1.27E-04 | 7.18 |
|  | glutathione metabolic process | 1.27E-04 | 7.18 |
|  | coenzyme metabolic process | 2.08E-04 | 2.85 |
|  | cofactor metabolic process | 2.12E-04 | 2.58 |
|  | glutathione biosynthetic process | 2.69E-04 | 13.41 |
|  | immune system process | 3.39E-04 | 1.55 |
|  | chromatin assembly or disassembly | 4.63E-04 | 2.90 |
|  | regulation of myeloid cell differentiation | 4.69E-04 | 3.83 |
|  | neuropeptide signaling pathway | 6.46E-04 | 3.24 |
|  | sulfur metabolic process | 7.34E-04 | 2.92 |
|  | regulation of cyclase activity | 9.32E-04 | 3.05 |
|  | chromatin organization | 9.43E-04 | 1.86 |
|  | response to oxidative stress | 1.00E-03 | 2.45 |
| M4 |  |  |  |
|  | mitochondrial transport | 3.76E-04 | 12.59 |
|  | protein transport | 7.11E-04 | 2.85 |
|  | establishment of protein localization | 7.53E-04 | 2.82 |
|  | intracellular transport | 9.36E-04 | 2.97 |
| M5 |  |  |  |
|  | organelle organization | 1.20E-04 | 1.97 |
|  | protein ubiquitination | 6.42E-04 | 5.08 |
|  | protein modification by small protein conjugation | 9.83E-04 | 4.58 |
| M6 |  |  |  |
|  | organelle organization | 3.21E-04 | 1.84 |
|  | protein targeting | 7.83E-04 | 3.50 |
|  | protein targeting to peroxisome | 9.27E-04 | 20.17 |
| M7 |  |  |  |
|  | tissue morphogenesis | 2.57E-04 | 4.30 |
|  | morphogenesis of an epithelium | 3.84E-04 | 5.74 |
|  | primary neural tube formation | 4.62E-04 | 11.72 |
|  | epithelial tube morphogenesis | 4.96E-04 | 7.22 |
|  | coenzyme metabolic process | 4.98E-04 | 4.42 |
|  | macromolecular complex subunit organization | 6.32E-04 | 2.18 |
|  | neural tube formation | 7.94E-04 | 9.67 |
|  | macromolecular complex assembly | 8.51E-04 | 2.18 |
|  | cellular component assembly | 9.52E-04 | 1.96 |
|  | embryonic epithelial tube formation | 9.70E-04 | 8.99 |
| M8 |  |  |  |
|  | nucleosome organization | 3.15E-11 | 9.81 |
|  | nucleosome assembly | 9.65E-11 | 10.14 |
|  | chromatin assembly | 1.51E-10 | 9.79 |
|  | chromatin assembly or disassembly | 2.36E-10 | 7.67 |
|  | protein-DNA complex assembly | 2.67E-10 | 9.36 |
|  | DNA packaging | 6.89E-10 | 7.80 |
|  | macromolecular complex assembly | 4.02E-09 | 3.02 |
|  | cellular macromolecular complex assembly | 6.11E-09 | 4.21 |
|  | cellular macromolecular complex subunit organization | 9.86E-09 | 3.92 |
|  | macromolecular complex subunit organization | 1.81E-08 | 2.83 |
|  | cellular component assembly | 7.91E-07 | 2.33 |
|  | chromatin organization | 1.49E-06 | 3.22 |
|  | cellular component biogenesis | 1.56E-06 | 2.19 |
|  | chromosome organization | 4.86E-06 | 2.76 |
|  | cellular component organization | 3.23E-05 | 1.53 |
|  | organelle organization | 4.87E-05 | 1.78 |
|  | protein complex assembly | 3.96E-04 | 2.17 |
|  | protein complex biogenesis | 3.96E-04 | 2.17 |
|  | DNA integration | 4.01E-04 | 12.17 |
| M9 |  |  |  |
|  | cellular component biogenesis | 1.22E-05 | 2.76 |
|  | macromolecular complex assembly | 1.54E-05 | 3.28 |
|  | antigen processing and presentation  of peptide antigen via MHC class I | 1.93E-05 | 34.24 |
|  | antigen processing and presentation | 2.50E-05 | 10.52 |
|  | macromolecular complex subunit organization | 3.03E-05 | 3.07 |
|  | antigen processing and presentation  of peptide antigen | 8.83E-05 | 20.79 |
|  | cellular component assembly | 9.12E-05 | 2.63 |
|  | protein complex assembly | 2.25E-04 | 3.17 |
|  | protein complex biogenesis | 2.25E-04 | 3.17 |
|  | cellular macromolecular complex subunit organization | 2.97E-04 | 3.67 |
|  | nucleosome organization | 3.70E-04 | 7.82 |
|  | cellular macromolecular complex assembly | 5.94E-04 | 3.66 |
|  | ubiquitin-dependent protein catabolic process | 6.13E-04 | 4.21 |
|  | protein ubiquitination | 8.79E-04 | 6.11 |
|  | negative regulation of protein modification process | 8.79E-04 | 6.11 |

M is short for Module_
