# Supplementary material for: Discovering Cooperative Relationships of Chromatin Modifications in Human T Cells Based on a Proposed Closeness Measure
Source: PLoS One. 2010 Dec 3;5(12):e14219. doi: 10.1371/journal.pone.0014219 (PMC2997069; doi:10.1371/journal.pone.0014219)
Supplement: Table S5 — Potential between-feature interactions (Occurrence >1). (0.05 MB DOC) [file pone.0014219.s005.doc]

**Table S5 Potential between-feature interactions (Occurrence > 1). This table omits obvious interplays linking histone methylation modifications with different methylation number (such as H3K4me1-H3K4me2) or histone acetylation-acetylation interplays on distinct loci.**

| Feature | Feature | Occurrence |
| --- | --- | --- |
| H3K4me1 | H4K20me1 | 8 |
| H3K4me3 | PolII | 7 |
| H2BK5me1 | H3K4me1 | 6 |
| H2A.Z | H3K4me3 | 6 |
| H2A.Z | H3K18ac | 6 |
| H2BK5me1 | H4K20me1 | 5 |
| H3K4me3 | H3K18ac | 5 |
| H2A.Z | H3K4me2 | 4 |
| CTCF | PolII | 4 |
| PolII | H3K9ac | 4 |
| H2A.Z | H4K8ac | 4 |
| H2A.Z | H4K20me1 | 3 |
| H3K79me1 | H4K20me1 | 3 |
| H3K4me1 | PolII | 3 |
| H3K4me3 | H4K91ac | 3 |
| PolII | H2BK20ac | 3 |
| H3K4me3 | H3K9ac | 3 |
| H3K4me1 | H3K18ac | 3 |
| CTCF | H2A.Z | 3 |
| H3K4me1 | H3K79me1 | 2 |
| H3K79me2 | H4K20me1 | 2 |
| H4K20me1 | PolII | 2 |
| H3K4me1 | H4K91ac | 2 |
| H3K4me2 | H3K18ac | 2 |
