# Supplementary material for: Discovering Cooperative Relationships of Chromatin Modifications in Human T Cells Based on a Proposed Closeness Measure
Source: PLoS One. 2010 Dec 3;5(12):e14219. doi: 10.1371/journal.pone.0014219 (PMC2997069; doi:10.1371/journal.pone.0014219)
Supplement: Table S6 — Partial correlation coefficients for chromatin modifications in GM12878 and Hsmm. (0.05 MB DOC) [file pone.0014219.s006.doc]

**Table S5** Partial correlation coefficients for chromatin modifications in GM12878 and Hsmm.

In addition to literature support, the discovered cooperative relationships of chromatin modifications can be indirectly supported by partial correlation regression coefficients. High correlation does not mean causal relationships, which is well known to all. We carried out a partial correlation analysis to clarify the novel relationships which have not been validated by experiments and can therefore show the usefulness of our approach.

Partial correlation analysis is important when considering causal relationships between more than two features. Because the correlation between two features may overstate or understate the true relationship and others may have influences upon these two features. There are several studies that used the partial correlation analysis to generate causal relationships [1,2]. The concept in partial correlation analysis is the partial correlation coefficient r_xy,z_ between variables x and y, adjusted for other variables z (a 31237 by 19 matrix, reverse of x and y in *CM* profile). Both x and y are presumed to be linearly related to z:

x = Az + B + d_x_ (1)

y = Cz + D + d_y_ (2)

The partial correlation coefficient r_xy,z_ is defined as the correlation coefficient between residuals d_x_ and d_y_. The partial correlation coefficient r_xy.z_ between x and y adjusted for z can be computed from the pairwise values of the correlation between variables x , y , and z (r_xy_, r_yz_, r_xz_):

$r_{xy,z}=\frac{r_{xy}-r_{xz}r_{yz}}{\sqrt{(1-r_{xz}^{2})(1-r_{yz}^{2})}}$ (3)

The r_xy,z_ takes on values between -1 and 1.

The R function pcor.test downloaded from http://www.yilab.gatech.edu/pcor.html was used to do this analysis. The command to execute pcor.test is pcor.test(data [,arg(*A_j_*)],data [,arg(*A_k_*)],data [,-c(arg(*A_j_*), arg(*A_k_*)]), where arg represents the column index (start from 1) and *j*≠*k*.

We used ChIP-seq histone modifications datasets to infer the causal relationships. Two cell types were used, that is GM12878 (lymphoblastoid) and Hsmm (normal human skeletal muscle myoblasts). The tag data (only replication 1) was download from ENCODE (http://hgdownload.cse.ucsc.edu/goldenPath/hg18/encodeDCC/wgEncodeBroadChipSeq/). Only control, CTCF, H3K4me1/2/3, H3K9ac, H3K36me3, H4K20me1 enrichment were used. Other modifications were not used for they are not methylation-influential informative features. The data were preprocessed as follows: (1) Only gene environment regions of all RefSeq transcripts (TSS -10k ~ TES 1k) was selected, the overlapping regions were merged. (2) We partitioned the merged region into 500-bp bins with 200bp steps forward; (3) The 500bp bins were mapped by histone modification tags, the tag counts for all histone modifications form the tag profile; (3) The number of reads can be variable between different histone marks. For example, there are 14.9 million reads spanning H3K4me1 but only 10.0 million spanning H3K4me2 in GM12878. This vast difference in tag number makes it hard to compare different histone mark enrichment by comparing tag counts. To address the problem, we normalized the number of reads in each bin by the total tag number for each modification by the total tag number for the control data. For example, there are 7.9 million tags in control data, so the ratio of 7.9 and 14.9 is multiplied to each tag number for each bin of H3K4me1. After preprocessing, the adjusted tag number for each bin was used for further partial correlation analysis.

GM12878

| Partial correlation coefficient | CTCF | H3K4me1 | H3K4me2 | H3K4me3 | H3K9ac | H4K20me1 | H3K36me3 |
| --- | --- | --- | --- | --- | --- | --- | --- |
| CTCF | - | -0.09 | -0.08 | -0.07 | 0.12 | 0.06 | -0.02 |
| H3K4me1 | - | - | 0.53 | -0.12 | -0.07 | 0.11 | 0.14 |
| H3K4me2 | - | - | - | 0.23 | 0.32 | 0.13 | 0.00 |
| H3K4me3 | - | - | - | - | 0.42 | 0.12 | 0.06 |
| H3K9ac | - | - | - | - | - | 0.01 | -0.09 |
| H4K20me1 | - | - | - | - | - | - | 0.27 |
| H3K36me3 | - | - | - | - | - | - | - |

Hsmm

| Partial correlation coefficient | CTCF | H3K4me1 | H3K4me2 | H3K4me3 | H3K9ac | H4K20me1 | H3K36me3 |
| --- | --- | --- | --- | --- | --- | --- | --- |
| CTCF | - | -0.02 | -0.08 | -0.14 | 0.22 | 0.05 | -0.01 |
| H3K4me1 | - | - | 0.35 | -0.21 | -0.05 | 0.15 | 0.11 |
| H3K4me2 | - | - | - | 0.19 | 0.41 | 0.10 | 0.05 |
| H3K4me3 | - | - | - | - | 0.29 | 0.10 | -0.03 |
| H3K9ac | - | - | - | - | - | -0.01 | 0.05 |
| H4K20me1 | - | - | - | - | - | - | 0.36 |
| H3K36me3 | - | - | - | - | - | - | - |

The orange cell in Tables means that it is discovered in this work.

References

1. Freudenberg J, Wang M, Yang Y, Li W (2009) Partial correlation analysis indicates causal relationships between GC-content, exon density and recombination rate in the human genome. BMC Bioinformatics 10 Suppl 1: S66.

2. Opgen-Rhein R, Strimmer K (2007) From correlation to causation networks: a simple approximate learning algorithm and its application to high-dimensional plant gene expression data. BMC Syst Biol 1: 37.
